# Immediate versus staged revascularisation of non-culprit arteries in patients with acute coronary syndrome: a systematic review and meta-analysis

**DOI:** 10.1007/s12471-022-01687-7

**Published:** 2022-05-10

**Authors:** P. A. Vriesendorp, J. M. Wilschut, R. Diletti, J. Daemen, I. Kardys, F. Zijlstra, N. M. Van Mieghem, J. Bennett, G. Esposito, M. Sabate, W. K. den Dekker

**Affiliations:** 1 Department of Cardiology, Thoraxcenter, Erasmus University Medical Centre, Rotterdam, The Netherlands; 2 The Heart Centre, The Alfred Hospital, Melbourne, Australia; 3 Department of Cardiovascular Medicine, University Hospitals Leuven, Leuven, Belgium; 4 Division of Cardiology, Department of Advanced Biomedical Sciences, University of Naples Federico II, Naples, Italy; 5 Cardiovascular Institute, Hospital Clinic, IDIBAPS, University of Barcelona, Barcelona, Spain

**Keywords:** Multivessel coronary artery disease, Percutaneous coronary intervention, Acute coronary syndrome

## Abstract

Although there is robust evidence that revascularisation of non-culprit vessels should be pursued in patients presenting with an acute coronary syndrome (ACS) and multivessel coronary artery disease (MVD), the optimal timing of complete revascularisation remains disputed. In this systematic review and meta-analysis our results suggest that outcomes are comparable for immediate and staged complete revascularisation in patients with ACS and MVD. However, evidence from randomised controlled trials remains scarce and cautious interpretation of these results is recommended. More non-biased evidence is necessary to aid future decision making on the optimal timing of complete revascularisation.

**Supplementary Information:**

The online version of this article (10.1007/s12471-022-01687-7) contains supplementary material, which is available to authorized users.

## Introduction

In the majority of patients presenting with acute coronary syndromes (ACS), percutaneous coronary intervention (PCI) is the preferred modality of reperfusion [1, 2]. Up to 60% of patients presenting with ST-segment elevation myocardial infarction (STEMI) and non-ST-segment elevation acute coronary syndrome (NSTE-ACS) have multivessel coronary artery disease (MVD) on coronary angiography [3, 4]. Patients with MVD have a worse prognosis compared with patients with single-vessel disease [3, 4]. Both the 2017 European Society of Cardiology STEMI and the recently published 2020 NSTE-ACS guidelines encourage complete revascularisation in patients presenting with MVD, class IIa, level of evidence (LOE) A and C respectively [2, 5]. The recommendation in the STEMI guidelines was based on several randomised controlled trials (RCTs), which demonstrated that complete revascularisation is superior to a culprit-only strategy in terms of major adverse cardiovascular events (MACE), but the beneficial effect was driven by the need for revascularisation and reduction in angina [6–9]. The recently published COMPLETE trial was the first to demonstrate the superiority of complete revascularisation with respect to the primary endpoint of myocardial infarction (MI) or cardiovascular mortality [10].

Complete multivessel revascularisation seems the preferred strategy, but timing remains unclear. Complete revascularisation can be performed during the index procedure, after treatment of the culprit lesion. Alternatively, operators can perform culprit-only revascularisation during the index procedure, and staged complete revascularisation, either during index hospitalisation or even in an ambulatory setting. The STEMI guidelines do not contain recommendations on the timing of revascularisation, as data are limited: there are only three old and small RCTs in STEMI patients (*n* = ~300 patients). Based upon the SMILE study, the NSTE-ACS guidelines state that complete revascularisation during the index procedure may be considered, class IIB, LOE B [2, 5].

This systematic review and meta-analysis compares immediate complete revascularisation during the index procedure versus staged complete revascularisation in patients presenting with ACS (including STEMI and NSTE-ACS) and MVD. Only studies in which both immediate and staged complete revascularisation were compared were included. Because of the limited data, this systematic review included both RCTs and non-randomised trials.

## Methods

### Search strategy and study selection

The protocol for this systematic review and meta-analysis was developed and registered in the PROSPERO database (CRD42019124604) and was reported in accordance with the PRISMA statement guidelines [11]. The search strategy was developed using the main parameters (Table 1), and PubMed, EMBASE, Cochrane Library and MEDLINE online databases were searched for publications between 2000 and 1 April 2020. An example of the search strategy for EMBASE can be found in Table S1 (Electronic Supplementary Material). Pre-defined inclusion criteria were based on the main parameters and included head-to-head comparisons of immediate revascularisation of non-culprit arteries with staged revascularisation in patients with ACS and MVD. Both RCTs and non-randomised comparisons were included. The records obtained were assessed on the title and abstract and were excluded if one of the following was applicable: case reports, observational studies without a comparison group, reviews, meta-analyses, lack of relevant outcomes or study question and conference abstracts. Selected full text records were analysed by two independent reviewers (WKD and PAV) to determine if the inclusion criteria were fulfilled. Any disagreements were resolved by consensus. If > 1 publication was based on the same cohort or population and reported the same outcomes, only the most recent or comprehensive publication was included. In addition, a manual reference search of relevant literature was performed to ensure completeness.

**Table 1:** Main parameters of the systematic review

Patient population	Patients with all types of acute coronary syndrome and multivessel coronary artery disease
Intervention	Immediate revascularisation of non-culprit arteries
Comparator	Staged revascularisation of non-culprit arteries
Outcomes	30-day all-cause mortality 1‑year all-cause mortality Non-fatal myocardial infarction Non-fatal stroke Unplanned revascularisation Major cardiac adverse events
Study design	Randomised controlled trials (Propensity) matched cohorts Non-randomised non-matched comparisons

### Data extraction

A standardised and pre-piloted form was used for extracting data of the studies included. The following study characteristics were collected: age, sex, cardiac risk factors (diabetes mellitus, hypertension, smoking, hypercholesterolaemia, family history of premature cardiovascular disease), type of ACS (STEMI/NSTEMI/unstable angina), localisation of MI, and two- or three-vessel disease. The pre-specified primary outcomes collected were 30-day and 1‑year all-cause mortality. Secondary outcomes were unplanned revascularisation, MI, disabling stroke and MACE. For completeness, we also tried to contact authors via e‑mail or telephone to obtain further information that had not been reported in their published articles.

### Risk of bias assessment

After data extraction, quality assessment of each included record was performed. For RCTs, the risk of bias tool 2.0 [12] and for non-randomised analyses the ROBINS-I tool was used [13]. Risk of bias was assessed per study per domain. The distributions of (small study) treatment effects were evaluated in comparison with the obtained pooled effect using funnel plots. Asymmetrical funnel plots were further evaluated to determine if asymmetry was a result of selective outcome reporting or publication bias, or due to poor methodological quality, true heterogeneity or chance.

### Qualitative and statistical analysis

Data were included in the meta-analysis if there was no critical risk of bias. For each study included, treatment effect was reported as odds ratio (OR) with 95% confidence intervals (CI) for both primary and secondary outcomes. Heterogeneity was assessed visually and between-study heterogeneity using the *I*^2^ statistic. If collected data were considered appropriate for data synthesis based on the risk of bias assessment, a random-effects model was used, with the Sidik-Jonkman estimator for τ to estimate the between-study variance. Statistical analysis was performed using R (R Development Core Team, Vienna, Austria) with meta and metafor packages, and STATA 15.1 (StataCorp, College Station, TX, USA) with metan and confunnel packages.

## Results

### Study selection and characteristics

The results of the search process are presented in a PRISMA flow chart (Fig. S1, Electronic Supplementary Material), and after applying exclusion criteria 20 studies were selected for the systematic review [14–33]. A total of 10,737 patients were included: 4835 (45%) patients underwent immediate revascularisation of non-culprit arteries and 5902 (55%) staged revascularisation. Five RCTs were included but accounted for only 916 (9%) patients [15, 18, 20, 25, 28]. Most patients (9116, 85%) presented with a STEMI. Patients presenting with cardiogenic shock at the time of the index PCI were not excluded in 6 studies [14, 16, 17, 19, 21, 26] and in 4 of these studies significantly more patients with cardiogenic shock were included in the immediate complete revascularisation group [14, 16, 17, 19]. An overview of individual study and baseline characteristics is shown in Table 2.

**Table 2:** Study characteristics

Study	Study type	ACS	Patients, *n*		Age, years		Female		Diabetes mellitus		Anterior MI		Three-vessel disease		CS excluded
			IR	SR	IR	SR	IR	SR	IR	SR	IR	SR	IR	SR	
Corpus et al. 2004 [14]	Non-randomised	STEMI	26	126											No^a^
Ochala et al. 2004 [15]	RCT	STEMI	48	44	65	67	27	25	31	34	46	45			Yes
Varani et al. 2008 [16]	Non-randomised	STEMI	147	96	69	67	33	32			48	33	35	47	No^a^
Hannan et al. 2010 [17]	Non-randomised	STEMI	503	538			23	23	24				26	35	No^a^
Politi et al. 2010 [18]	RCT	STEMI	65	65	65	64	23	20	14	18	48	49	29	45	Yes
Kornowski et al. 2011 [19]	Non-randomised	STEMI	275	393	62	64	24	19	15	18	41	35			No^a^
Maamoun et al. 2011 [20]	RCT	STEMI	42	36	55	52	5	11	40	56	62	69	26	22	Yes
Mohamad et al. 2011 [21]	Non-randomised	STEMI	7	12											No
Jensen et al. 2012 [22]	Non-randomised	STEMI	354	820	65	65	24	20	11	10	48	37	34	61	Yes
Kim et al. 2014 [23]	Non-randomised	STEMI	67	252	66	69									Yes
Manari et al. 2014 [24]	Non-randomised	STEMI	367	988	67	66	30	21	19	18	47	35	11	15	Yes
Tarasov et al. 2014 [25]	RCT	STEMI	46	43	59	59	30	42	26	21	46	30	43	47	Yes
Chung et al. 2016 [26]	Non-randomised	STEMI	66	41	64	62	32	22			62	46			No
Khan et al. 2016 [27]	Non-randomised	STEMI	63	30	63	65	29	7	17	13	32	37			Yes
Sardella et al. 2016 [28]	RCT	NSTEMI	264	263	72	73	22	21	37	40					Yes
Yu et al. 2016 [29]	Non-randomised	NSTEMI	420	420	63	63	71	73	38	38			59	58	Yes
Iqbal et al. 2017 [30]	Non-randomised	STEMI	1325	658	65	64	19	23	28	21	23	35			Yes
Kim et al. 2017 [31]	Non-randomised	STEMI	316	437	62	63	24	28	36	32	50	36	39	42	Yes
Doğan et al. 2019 [32]	Non-randomised	Mixed	180	425	59	58	28	90	49	108	48	99			Yes
Tovar Forero et al. 2020 [33]	Non-randomised	STEMI	254	215	66	62	29	22	13	12	36	29	22	38	Yes

All variables are expressed as a percentage, unless stated otherwise

*ACS* acute coronary syndrome, *CS* cardiogenic shock, *IR* immediate revascularisation, *MI* myocardial infarction, *NSTEMI* non-ST-elevated myocardial infarction, *RCT* randomised controlled trial, *STEMI* ST-elevated myocardial infarction, *SR* staged revascularisation (either in-hospital or after index admission)

^a^Significantly higher number of patients with CS in direct complete revascularisation

### Risk of bias

**Fig. 1:**
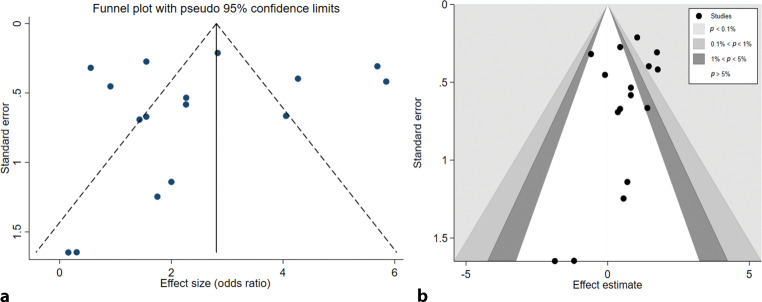
Funnel plot (**a**) and contour-enhanced funnel plot (**b**). There is asymmetry, most likely due to heterogeneity. Publication bias seems unlikely due to the location of the evidence gap and no suggestion of missing studies in the area of non-significance

Risk of bias was assessed for all individual studies. No studies were considered at critical risk of bias, but a substantial risk of bias was present in all non-randomised studies. Several non-randomised studies used propensity score matching to reduce confounding [17, 19, 24, 26, 29, 30, 32], but even in these studies a risk of residual confounding remained because of the inability to correct for unobserved factors. Funnel-plot analyses demonstrated an asymmetrical distribution, most likely due to heterogeneity of the studies included. Publication bias seemed unlikely, due to the location of the evidence gap: there was no suggestion of missing studies in the area of non-significance (Fig. 1a,b).

### Clinical outcomes

**Fig. 2:**
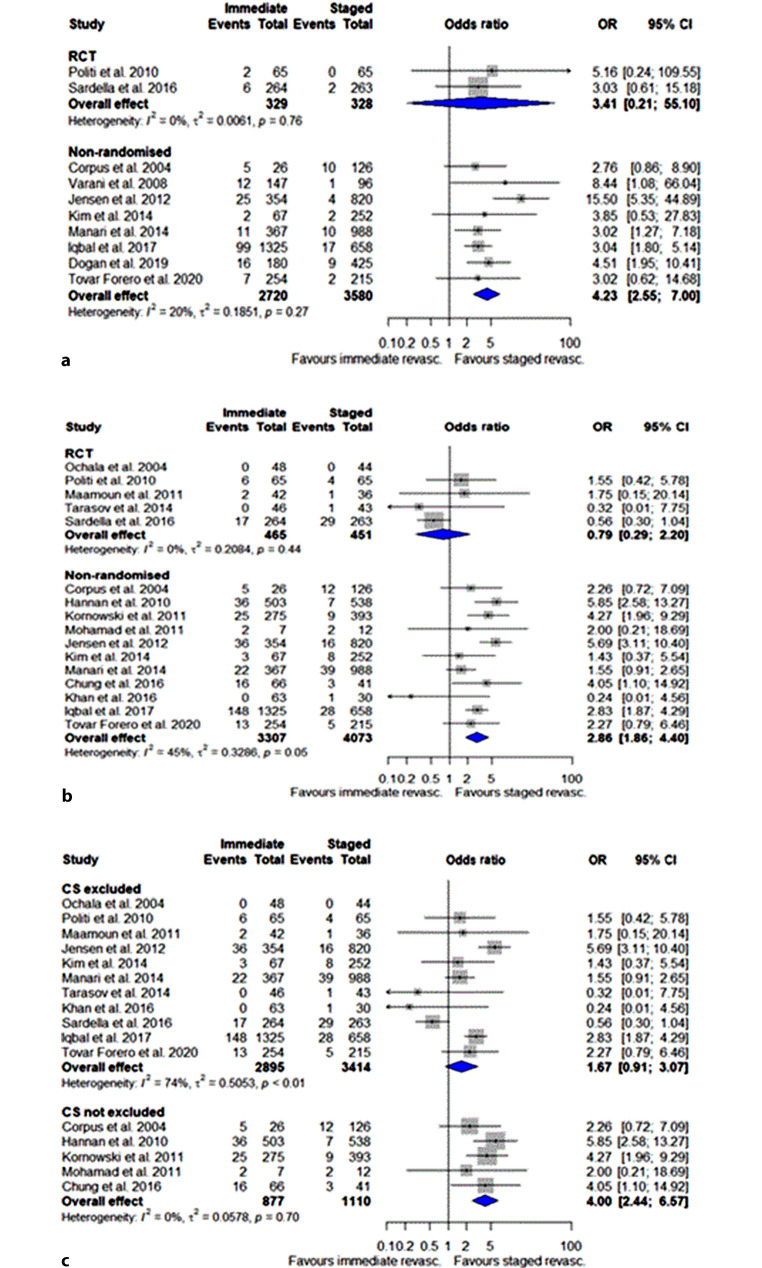
Thirty-day (**a**) and 1‑year (**b**) mortality risk of patients with acute coronary syndrome who underwent immediate or staged revascularisation of non-culprit arteries. **c** One-year mortality risk of patients with acute coronary syndrome who underwent immediate or staged revascularisation of non-culprit arteries with or without exclusion of patients in cardiogenic shock (*CS*) (*RCT* randomised controlled trail, *OR* odds ratio, *CI* confidence interval)

Ten studies (6957 patients, 65%; 2 RCTs and 8 non-randomised studies) reported on 30-day mortality outcomes and the data were suitable for data synthesis to determine the 30-day mortality risk. The 30-day mortality risk was comparable in the RCTs (OR 3.4, 95% CI 0.2–55.1), but this was based on 2 studies with a limited number of events (Fig. 2a). In the non-randomised studies, the risk appeared to be significantly higher for patients who underwent immediate revascularisation of non-culprit arteries compared with patients who underwent staged revascularisation (OR 4.2, 95% CI 2.6–7.0), as is shown in Fig. 2a.

Sixteen studies (8296 patients, 77%) were included in the data synthesis to determine the 1‑year mortality risk. Of these, 11 studies were non-randomised and 5 studies were RCTs (7380 patients and 916 patients respectively). One-year mortality was comparable between immediate and staged complete revascularisation in the RCTs (OR 0.8, 95% CI 0.3–2.2), and was increased for patients that underwent immediate complete revascularisation in the non-randomised trials (OR 2.9, 95% CI 1.9–4.4; Fig. 2b).

However, if studies that included patients with cardiogenic shock were excluded, 1‑year mortality was comparable with both strategies (OR 1.7, 95% CI 0.9–3.1; Fig. 2c). There was no difference between immediate and staged revascularisation as regards the risk of unplanned revascularisation: OR 0.88 (95% CI 0.52–1.47) in non-randomised studies and OR 0.71 (95% CI 0.26–1.95) in randomised studies (see Figure S 3a in Electronic Supplementary Material [ESM]) or MI: OR 0.91 (95% CI 0.54–1.54) in non-randomised studies and OR 0.92 (95% CI 0.23–3.61) in randomised studies (see Figure S 3b in Electronic Supplementary Material [ESM]). There were insufficient data to compare the two strategies regarding other outcomes such as stroke, urgent revascularisation or MACE.

## Discussion

In this systematic review and meta-analysis, we compared immediate complete revascularisation with staged complete revascularisation in patients with ACS and MVD. In a pooled analysis of the RCTs there was no difference in 30-day and 1‑year mortality between the two revascularisation strategies. In non-randomised studies, mortality was higher in patients who underwent immediate complete revascularisation compared to staged complete revascularisation. However, these registries were prone to important confounding bias, as more patients in cardiogenic shock underwent ad hoc complete revascularisation. Indeed, the increased mortality risk was no longer present when studies that also allowed cardiogenic shock were excluded.

There is compelling evidence that complete revascularisation rather than culprit-only revascularisation is beneficial among patients with STEMI and MVD [6–10]. The COMPLETE trial showed a clear benefit of complete revascularisation in terms of prognostically relevant clinical endpoints (mortality and MI). Nevertheless, the optimal timing of complete revascularisation remains disputed. Complete revascularisation can be achieved during the index procedure or in a staged fashion during index hospitalisation or even after discharge. Immediate complete revascularisation prevents exposure to a second invasive procedure with all its associated risks and costs. Conversely, in the index procedure there might be an overestimation of the severity of non-culprit lesions due to higher vascular tone, a higher risk of stent thrombosis and peri-procedural MI due to the prothrombotic milieu, a higher risk of contrast nephropathy due to the use of more contrast and a higher risk of arrhythmia. The COMPLETE timing sub-study showed that the benefit of complete revascularisation was irrespective of whether complete revascularisation was performed during index hospitalisation or after discharge [34]. Notably, the trial did not allow complete revascularisation during the index procedure.

Data comparing the timing of complete revascularisation in ACS patients with MVD are ambiguous and RCTs are scarce. Although meta-analyses have been performed previously, they all used different inclusion criteria and outcomes than ours. In particular, a direct comparison between immediate complete revascularisation and staged complete revascularisation is lacking, as most meta-analyses also included studies that compared immediate or staged complete revascularisation with culprit-only revascularisation and subsequently performed network or pairwise analyses. Vlaar et al. performed a pairwise and network meta-analysis including 4 prospective and 14 retrospective studies [35]. They found that immediate complete multivessel primary PCI for STEMI was associated with the highest mortality rates for both short-term and long-term outcomes. These findings were confirmed by two subsequent meta-analyses by Tarantini et al. and Li et al., also showing an increased risk of mortality associated with immediate complete revascularisation [36, 37]. However, three meta-analyses including only RCTs showed no difference in mortality or MACE between immediate complete or staged complete revascularisation, but lower rates of recurrent MI in the immediate complete group [38–40]. Gaffar et al. did perform a meta-analysis that included only studies comparing immediate complete revascularisation and staged complete revascularisation in STEMI and NSTEMI patients [41]. However, they included only RCTs, so this yielded no more than 4 RCTs with a total of 853 patients. The risk of unplanned repeat revascularisation was significantly lower in the immediate complete group, while there was also a trend toward a lower risk of MACE.

In contrast to these earlier meta-analyses, we included both STEMI and NSTE-ACS patients, as well as RCTs and non-randomised studies. Moreover, we included studies up until April 2020 and only those in which both immediate and staged complete revascularisation were performed, creating a more homogeneous study population and allowing true head-to-head analysis.

Our meta-analysis revealed different outcome effects with immediate complete revascularisation in the RCTs as compared to the non-randomised studies. Cardiogenic shock was excluded from the RCTs but allowed in registries. Although complete revascularisation in ACS and MVD seems beneficial, this beneficial effect seems to be restricted to patients with ACS and MVD in the absence of cardiogenic shock [42]. The CULPRIT-SHOCK trial demonstrated that for patients in cardiogenic shock and with MVD at the time of acute MI, PCI of the culprit lesion only was superior to immediate complete revascularisation at 30 days, with a significantly lower mortality in the culprit-only PCI group [43]. For patients in cardiogenic shock, the longer procedure time and increased contrast volume do not outweigh the possible reduction in peri-infarct ischaemia or early recurrent ischaemia. Current guidelines do not advocate routine multivessel PCI in acute MI complicated by cardiogenic shock. An unequal or unknown distribution of patients with cardiogenic shock in non-randomised studies may present an important source of bias and could lead to unfair interpretation of results. As a matter of fact, when cardiogenic shock was excluded, there was also no difference in mortality between immediate complete revascularisation and staged revascularisation in the registries.

The unequal inclusion of cardiogenic shock patients illustrates the most important limitation of this meta-analysis: the shortage of data stemming from RCTs (916 patients, 8%). Non-randomised studies are always at an increased risk of bias, and although some studies reduced bias by performing propensity score matching [17, 19, 24, 26, 29, 30, 32] this cannot correct for unmeasurable confounders. Especially in patients presenting with STEMI, it is feasible that in retrospective analyses there were reasons not explicitly stated in the reports why operators preferred to perform complete revascularisation during the index procedure.

To bridge the noticeable evidence gap, we have initiated the BIOVASC trial (NCT03621501) [44]. In this study, all patients presenting with ACS (STEMI and NSTE-ACS) and MVD are being randomised to immediate complete revascularisation or culprit-only PCI plus staged revascularisation within 6 weeks after the index procedure. Significant coronary artery disease is defined as at least 70% stenosis in a vessel ≥ 2.5 mm by visual estimation or positive coronary physiology testing. Principal exclusion criteria are cardiogenic shock, no clear culprit lesion, prior coronary artery bypass surgery or the presence of a chronic total occlusion. The primary endpoint is a composite of death from any cause, MI, unplanned ischaemia-driven revascularisation or cerebrovascular events after 1‑year follow-up. Enrolment of 1525 patients was completed in October 2021 and the first results are expected at the end of 2022. BIOVASC aims to provide further insights into the clinical implications of immediate complete revascularisation across the entire ACS spectrum, to evaluate different effects in STEMI versus NSTE-ACS patients, and to explore the impact of the timing of revascularisation on early and late quality of life. The iMODERN (NCT03298659) and MULTISTARS AMI (NCT03135275) [45] studies are also investigating the timing of revascularisation, but there are some marked differences compared with the BIOVASC trial. iMODERN is a European multicentre trial that compares instantaneous wave-free (iFR)-guided immediate complete revascularisation with staged stress perfusion cardiac MRI-guided complete revascularisation (within 6 weeks after STEMI). Thus, unlike the BIOVASC trial, physiological assessment by iFR or cardiac MRI is mandatory. Furthermore, the iMODERN trial includes only STEMI patients, and those with complex bifurcation lesions and left main stenosis of ≥ 50% are excluded. Patients in cardiogenic shock are also excluded, as in the BIOVASC trial. A total of 1146 patients will be enrolled and the primary endpoint is a composite of all cause death, recurrent MI and hospitalisation for heart failure at 1 year. Enrolment is expected to be completed at the beginning of 2022. MULTISTARS AMI is also a European multicentre trial that compares immediate complete revascularisation with staged complete revascularisation in STEMI patients. The staged procedure has to be performed at least 19 days after the index procedure, but within 45 days. Lesions are considered significant if they cause a ≥ 70% diameter stenosis by visual estimation. Like the iMODERN study, patients with left main stenosis of ≥ 50% and those in cardiogenic shock are excluded. The primary endpoint is a composite of death, non-fatal MI, ischaemia-driven revascularisation hospitalisation for heart failure and stroke at 1 year. Although the anticipated number of patients to be enrolled was 840, this figure will probably not be reached as patient inclusion is planned only until the end of 2022, while after 3.5 years only 393 patients had been enrolled. The contribution of these three trials could help mitigate the limitations of this meta-analysis and aid future clinical decision-making.

In conclusion, this overview and meta-analysis suggests similar outcomes with an immediate or staged complete revascularisation strategy in patients with ACS and MVD without cardiogenic shock. However, these findings are mainly driven by non-randomised studies with a significant risk of bias and therefore support ongoing randomised trials on this topic to determine the optimal timing of complete revascularisation.

## Supplementary Information

Supplementary information containing search strategy and flow chart of study

Unplanned revascularisation (a) and myocardial infarction (b) risk of patients with acute coronary syndrome who underwent immediate or staged revascularisation of non-culprit arteries.
