# Supplementary material for: Immediate versus staged revascularisation of non-culprit arteries in patients with acute coronary syndrome: a systematic review and meta-analysis
Source: Neth Heart J. 2022 May 10;30(10):449–56. doi: 10.1007/s12471-022-01687-7 (PMC9474746; doi:10.1007/s12471-022-01687-7)

# Supplementary material

## Tables

**Table S1.** Search strategy and results in Ovid MEDLINE database.

| 1 | exp percutaneous coronary intervention/ | 48679 |
| --- | --- | --- |
| 2 | exp acute coronary syndrome/ or exp unstable angina pectoris/ or exp myocardial infarction/ | 182948 |
| 3 | ((staged or stage* or (stage* and revascularization) or (stage* and revascularisation)).tw.) or (((non-culprit or (non and culprit)) and (revasculari* or intervention*)).tw.) or (((multivessel and revasculari*) or (multi-vessel and revasculari*) or (multi* and revasculari*)).tw.) or (((multivessel or (multi* and vessel)) and (revasculari* or intervention* or disease)).tw.) | 999335 |
| 4 | 1 and 2 and 3 | 2710 |
| 5 | limit 4 to (english language and yr="2000 - 2020") | 2219 |

## Figures

**Figure S1.** PRISMA flowchart of the study selection process.


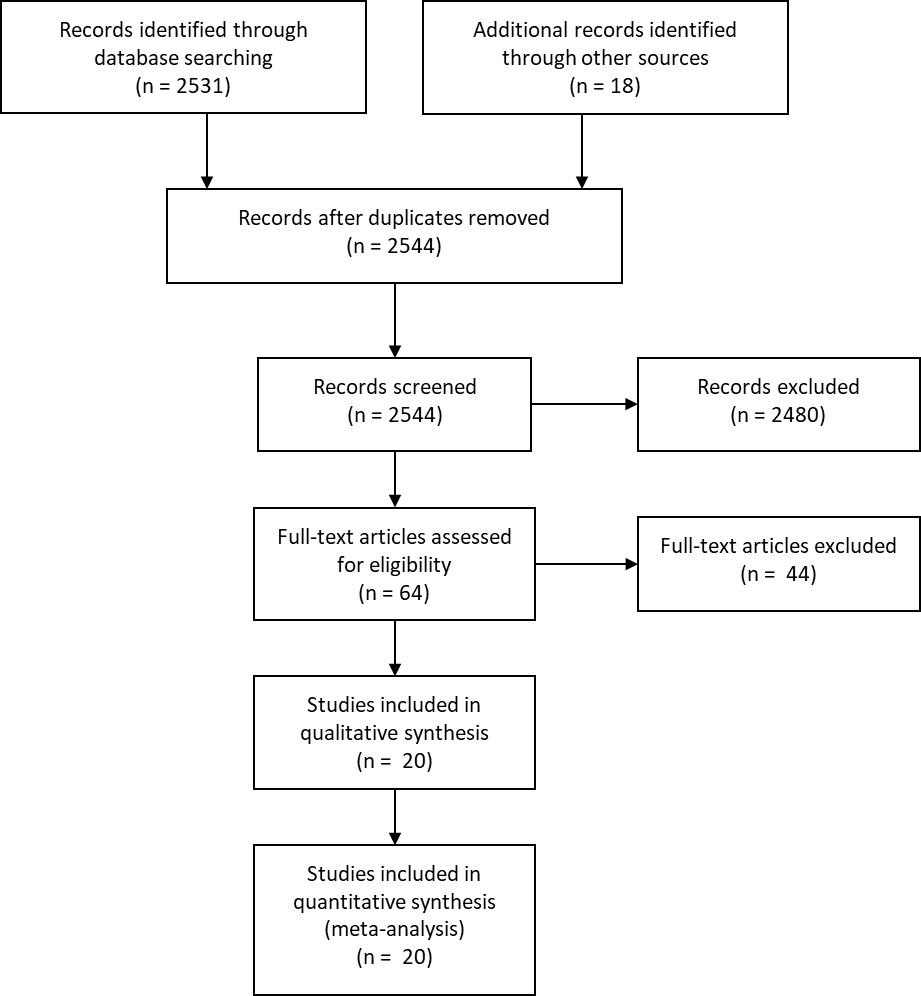


**VISUAL OVERVIEW** - This meta-analysis suggests similar outcomes with an immediate or staged complete revascularisation strategy in patients with acute coronary syndrome (ACS) and multivessel disease (MVD). In non-randomised studies, mortality was higher in patients who underwent immediate complete revascularisation compared to staged complete revascularisation. However, these registries were prone to important confounding bias as more patients in cardiogenic shock underwent ad hoc complete revascularization. Indeed, the increased mortality risk was no longer present when studies that also allowed cardiogenic shock were excluded.


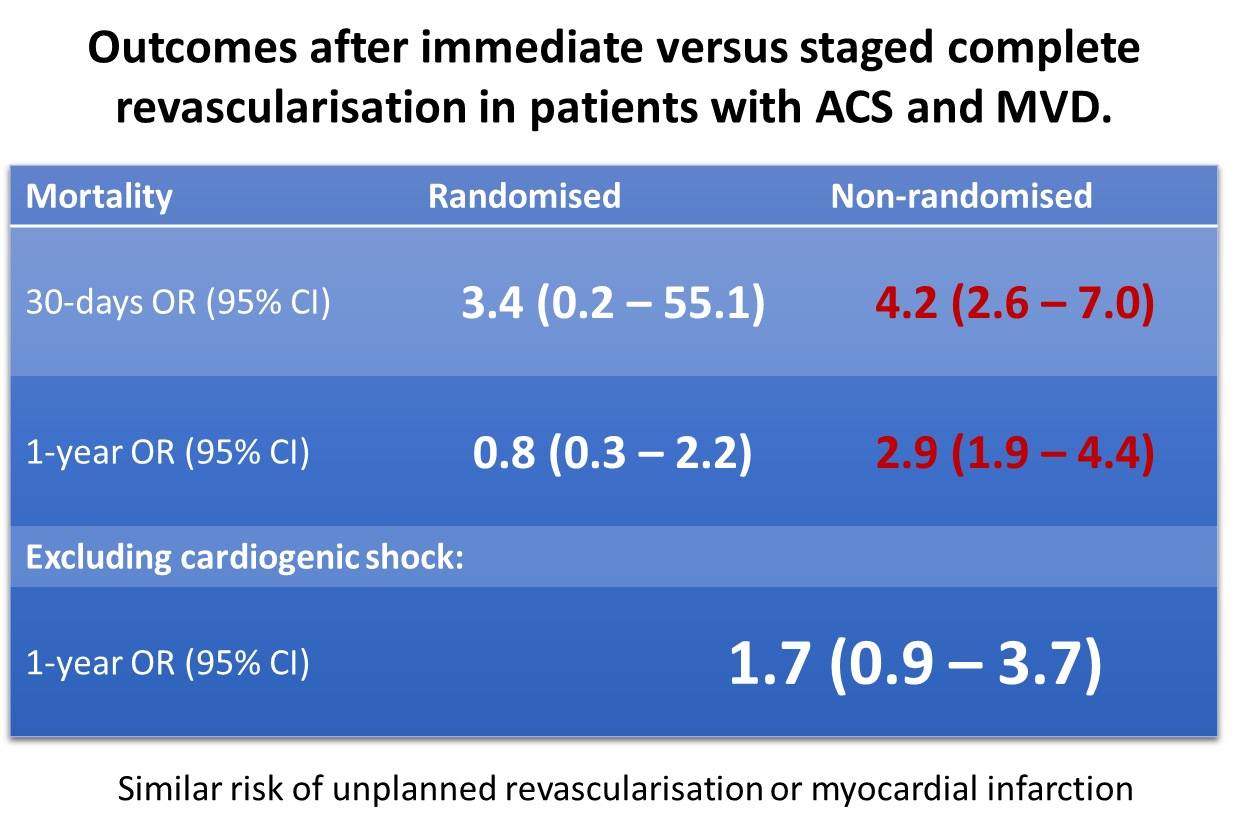

Supplement: Supplementary file 1 — Supplementary information containing search strategy and flow chart of study [file 12471_2022_1687_MOESM1_ESM.docx]
