# Supplementary figures and images for: Immediate versus staged revascularisation of non-culprit arteries in patients with acute coronary syndrome: a systematic review and meta-analysis
Source: Neth Heart J. 2022 May 10;30(10):449–56. doi: 10.1007/s12471-022-01687-7 (PMC9474746; doi:10.1007/s12471-022-01687-7)

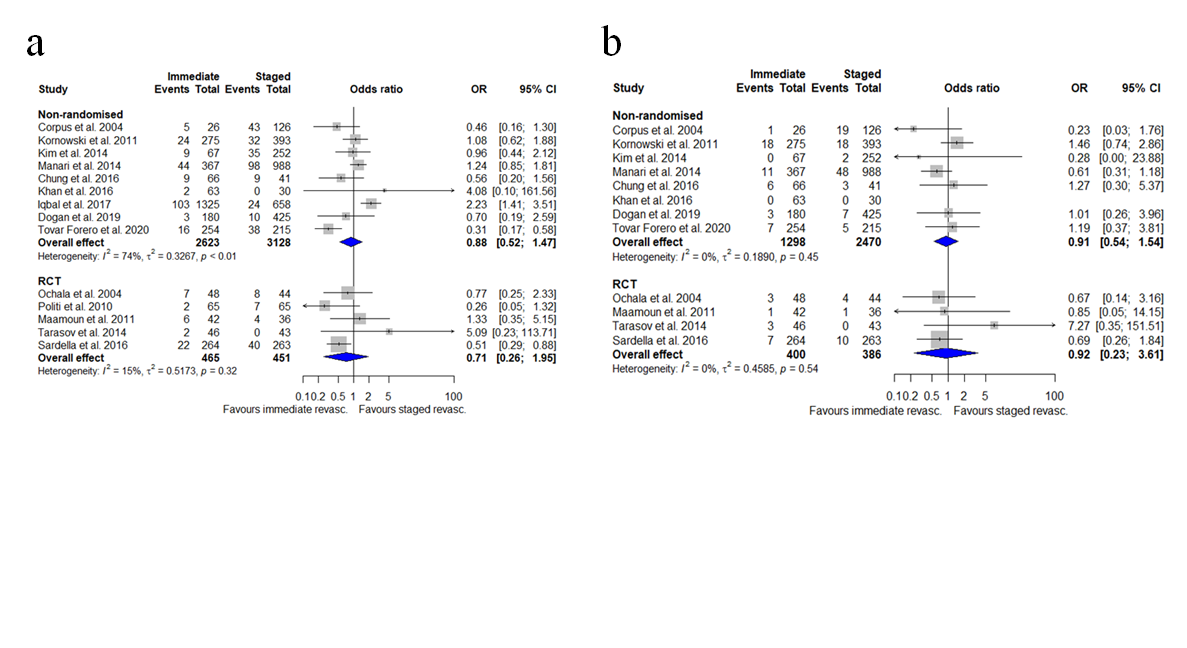

Supplement: Supplementary file 2 — Unplanned revascularisation (a) and myocardial infarction (b) risk of patients with acute coronary syndrome who underwent immediate or staged revascularisation of non-culprit arteries. [file 12471_2022_1687_MOESM2_ESM.tif]
